# Lipopolysaccharide stimulates egg laying in *Caenorhabditis elegans*

**DOI:** 10.17912/micropub.biology.000308

**Published:** 2020-09-14

**Authors:** Angela Ching-Yee Leung, Catherine Hughes, Jakob Gunderson, Myeongwoo Lee

**Affiliations:** 1 Institute of Biomedical Studies, Baylor University, Waco, TX 76798, U.S.A.; 2 Department of Biology, Baylor University, Waco, TX 76798, U.S.A.

**Figure 1. Wild-type N2 strain of C. elegans was stimulated by Salmonella enterica lipopolysaccharide (LPS) to lay eggs in an aqueous environment:**
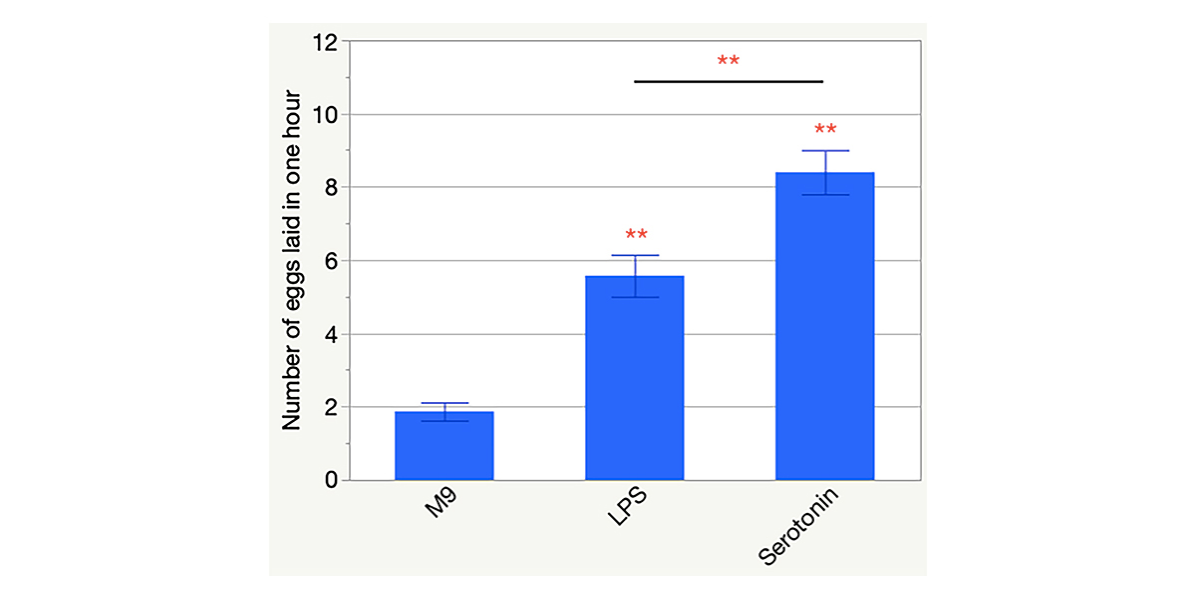
Animals were incubated in M9 buffer for 15 minutes, after which the numbers of eggs laid were recorded for adjustment purpose. The liquid medium was then supplemented with either LPS or serotonin, or with no supplement as the negative control. The final concentration of LPS was 0.1 mg/ml and that of serotonin was 1 mg/ml. After one hour, the eggs in each microtiter well were counted again. For each individual, the adjusted number of eggs laid, which considered only the eggs laid during the treatment period, is presented here. More than 100 animals under each condition were tested. Error bars represent standard errors; asterisks denotes p < 0.01 when the treatment condition (LPS or serotonin) resulted in an egg-laying level that was significantly different from that of the negative control condition (M9), when the non-parametric Mann-Whitney U Test (Mann and Whitney 1947) was performed. The horizontal bar and the asterisks above it indicate that there was a significant difference (p < 0.01, Mann-Whitney U Test; Mann and Whitney 1947) between the numbers of eggs laid under the LPS and serotonin conditions.

## Description

*Caenorhabditis elegans* is fed with the unharmful bacterial food of *Escherichia coli* strain OP50 in the laboratory but the nematode is also susceptible to infections by ingested Gram-negative bacterial pathogens. *Salmonella enterica* invades epithelial cells, causes germline cell death, and kills the worm in as short as two days, while *E. coli* OP50 does not result in any worm death for at least four days (Aballay *et al.* 2003; Tenor *et al.* 2004). When the worms are allowed to grow into 1-day-old adults on a lawn of *S. enterica*, the expression of an innate response gene, PMK-1, is activated, indicating infection by the pathogen and the elicited innate response of the host (Tenor *et al.* 2004). Here we report that lipopolysaccharide (LPS) extracted from *S. enterica* stimulated gravid adults to lay more eggs in LPS-containing solution than those in M9 buffer only. Figure 1 shows that the worms that were exposed to 0.1 mg/ml LPS for 1 hour laid an average of 5.57±0.57 eggs (± one standard error; *n* = 101), which was significantly different (*p* < 0.0001) than the response of the worms exposed to M9 buffer (1.87±0.25 eggs; *n* = 112). Serotonin, produced by the hermaphrodite-specific neurons (HSNs), is a known stimulant of the vulval muscle contractions that induce egg laying (Trent *et al.* 1983). Therefore, we used serotonin in this study as a positive control condition, and the egg-laying level that it induced as a reference to that caused by LPS. Exogenous serotonin at 1 mg/ml in M9 resulted in the laying of 8.40±0.60 eggs in one hour (*n* = 111; *p* < 0.0001 compared to the worms in M9). The magnitude of LPS-stimulated egg laying was not comparable to that of the serotonin one (*p* = 0.0005, indicating significant difference).

Intact LPS on the cell surface of *S. enterica* is required for *C. elegans* to initiate its innate immune response through the MAP kinase pathway involving PMK-1 (Aballay *et al.* 2003), while this study demonstrated that LPS extracted from the same bacterial species stimulated egg laying. Further studies will investigate whether LPS-stimulated egg laying involves activating the MAP kinase pathway and whether the stimulated egg-laying behavior happens simultaneously with the innate immune response in the nematode.

Since serotonin and the neuropeptide NLP-3 are released from the HSNs, to initiate egg-laying behavior (Brewer *et al.* 2019), further investigation is needed to understand whether the components of serotonin biosynthesis and signaling (Dempsey *et al.* 2005; Schafer 2006) or those of NLP-3 (which are yet to be identified), mediate LPS-stimulated egg laying. We speculate that LPS, when ingested, may stimulate egg laying by stimulating serotonin or neuropeptide production.

## Methods

Egg-laying assays were performed with a modified protocol from Trent *et al.* (1983). Independent assays were carried out at 21 to 23^o^C in microtiter plates. The microtiter wells were filled with 100 µl M9 buffer. One gravid wild-type animal, which was at larval stage 4 the day before and had been grown on a fresh OP50 lawn for 16 to 24 hours, was transferred to each well and allowed to lay eggs for 15 minutes, and the number of eggs was recorded prior to LPS or serotonin addition. Serotonin is a known egg-laying stimulant (Trent *et al.* 1983), therefore serving as the positive control condition in each independent assay. 2 µl of 5 mg/ml LPS in M9 or 5 µl of 20 mg/ml serotonin in M9 was then added to each well, such that the final concentration of LPS was 0.1 mg/ml and that of serotonin was 1 mg/ml. Unsupplemented M9 buffer served as the negative control condition. After 1 hour, the number of eggs in each well was observed again, and the difference was the number of eggs laid in 1 hour under the treatment or untreated condition. A total of over 100 animals under each of the three conditions were assayed in more than three independent trials. The distribution of the parameter, the number of eggs laid in one hour, of each condition, was found to be not normal. Therefore, the non-parametric Mann-Whitney test was carried out in JMP®PRO 14.0.0 (SAS Institute, Inc., Cary, NC, U.S.A.) to compare the number of eggs laid under each treatment condition to that under no treatment. The number of eggs laid as a result of the LPS treatment was also compared to that under the serotonin condition.

## Reagents

LPS from *Salmonella enterica* serotype typhimurium; Sigma L6511

Serotonin creatine sulfate complex; Sigma H7752

M9 buffer (Stiernagle 1999)

Nematode growth medium (NGM) agar plates (Brenner 1974)
